# O.6.1-3 The association between education outside the classroom, intrinsic school motivation, and physical activity in primary school pupils

**DOI:** 10.1093/eurpub/ckad133.262

**Published:** 2023-09-11

**Authors:** Silje Mikkelsen, Alberte Laura Oest Müllertz, Mads Bølling, Mette Aadahl, Peter Elsborg

**Affiliations:** Center for Clinical Research and Prevention, Copenhagen University Hospital – Bispebjerg and Frederiksberg, Nordre Fasanvej 57, 2000 Frederiksberg, Denmark; Center for Clinical Research and Prevention, Copenhagen University Hospital – Bispebjerg and Frederiksberg, Nordre Fasanvej 57, 2000 Frederiksberg, Denmark; Center for Clinical Research and Prevention, Copenhagen University Hospital – Bispebjerg and Frederiksberg, Nordre Fasanvej 57, 2000 Frederiksberg, Denmark; VIA University College, Research Centre for Pedagogy and Bildung, Hedeager 2, 8200 Aarhus N, Denmark; Center for Clinical Research and Prevention, Copenhagen University Hospital – Bispebjerg and Frederiksberg, Nordre Fasanvej 57, 2000 Frederiksberg, Denmark; Department of Clinical Medicine, Faculty of Health and Medical Sciences, University of Copenhagen, Denmark, Bispebjerg Bakke 23, 2400 Copenhagen NV, Denmark; silje.mikkelsen@regionh.dk; Center for Clinical Research and Prevention, Copenhagen University Hospital – Bispebjerg and Frederiksberg, Nordre Fasanvej 57, 2000 Frederiksberg, Denmark

## Abstract

**Purpose:**

The decline of school motivation over a school year is a common issue that can have negative impacts on academic achievement and children's health. Education outside the classroom (EOtC) has been shown to positively affect intrinsic school motivation and physical activity (PA) in previous studies. However, the relationship between motivation and PA during EOtC remains unclear. This study aims to investigate the association between EOtC and intrinsic school motivation and what role PA has in this association.

**Methods:**

Cross-sectional data from the Danish MOVEOUT study intervention group were collected from 225 primary school pupils aged 10-15 from 15 different school classes from September 2022 to February 2023. Intrinsic school motivation was measured by Intrinsic Motivation Inventory and Psychological Need Satisfaction Scales (BPNSS) items at pupil-level immediately after two EOtC and two non-EOtC schooldays. PA was measured with wrist band worn Axivity® AX3 accelerometers. Data was analysed with linear mixed models.

**Results:**

The study found a positive association between EOtC and higher levels of school motivation. The study also found associations between PA and intrinsic motivation, indicating that more time spent in sedentary behavior was associated with less school motivation, while more time in light intensity activity was associated with higher school motivation. However, the data did not support that PA moderates the association between EOtC and school motivation.

**Conclusion:**

The results of this study suggest that EOtC have a positive impact on pupil’s school motivation. We therefore recommend the use of EOtC to enhance intrinsic motivation. The study also highlights the importance of reducing sedentary behavior during school days. These findings can inform education policymakers and teachers in designing learning environments, lessons, and activities that promote pupils’ motivation and academic and personal growth.

**Funding:**

Novo Nordisk Foundation (Grant no. 0062703).

